# Hybrid prediction of infections and deaths due to COVID-19 in two Colombian data series

**DOI:** 10.1371/journal.pone.0286643

**Published:** 2023-06-08

**Authors:** Mónica Paola de la Cruz, Diana Milena Galvis, Gladys Elena Salcedo

**Affiliations:** Grupo de Investigación y Asesoría en Estadística, Universidad del Quindío, Armenia, Quindío, Colombia; Federal University of Pernambuco: Universidade Federal de Pernambuco, BRAZIL

## Abstract

The prediction of the number of infected and dead due to COVID-19 has challenged scientists and government bodies, prompting them to formulate public policies to control the virus’ spread and public health emergency worldwide. In this sense, we propose a hybrid method that combines the SIRD mathematical model, whose parameters are estimated via Bayesian inference with a seasonal ARIMA model. Our approach considers that notifications of both, infections and deaths are realizations of a time series process, so that components such as non-stationarity, trend, autocorrelation and/or stochastic seasonal patterns, among others, must be taken into account in the fitting of any mathematical model. The method is applied to data from two Colombian cities, and as hypothesized, the prediction outperforms the obtained with the fit of only the SIRD model. In addition, a simulation study is presented to assess the quality of the estimators of SIRD model in the inverse problem solution.

## 1 Introduction

COVID-19 is a highly infectious disease caused by the SARS-CoV-2 virus, first reported on 31 December 2019 in Wuhan, China. In early 2020, the disease crossed borders and due to its severity and the high rates of virus spread, on 11 March 2020, the World Health Organization (WHO) declared it a pandemic. Since then, a large part of the scientific community has been investigating the new coronavirus from different perspectives, seeking to control or prevent high infection and death rates. Particularly, it was necessary to develop and apply mathematical models to provide short-, medium- and long-term forecasts of the infection to enable governments to develop control strategies [1–3]. Different mathematical models based on nonlinear systems of ordinary differential equations (ODE), statistical models for time series and computational models, among others, have been used to describe the dynamics of the disease.

The most used mathematical approaches are the compartmental models, where the population is divided into states or sub-populations of Susceptible (S), Infected (I), Latent (L), Recovered (R), Vaccinated (V), Dead (D) and so on. In these models, the change from one state to another occurs through a transition rate and these rates, together with the basic reproductive number (*R*_0_) are denominated the model parameters, which can vary in function of the population, geographic location, sociocultural context, climate and control measures, among other factors. This type of models is based on differential equations systems and its solution can be approached from a direct or inverse perspective. From the direct perspective, given a model, initial conditions and a set of parameter values obtained from previous studies or at the discretion of the researcher, the number of individuals at each stage is predicted. This makes the predictions less accurate in comparison with real data. In turn, the inverse approach—using the official epidemiological data—estimates the model parameters through some statistical estimation method (e.g., classical or Bayesian inference) and then uses these values to solve the direct problem, obtaining a more realistic fit to the data observed [4, 5].

Solutions of the inverse problem in epidemic models through Bayesian inference have been widely used to study the behaviour of COVID-19. For example, [6] estimated the parameters of the SIR and SEIR models to study the COVID-19 in South Africa; [7] proposed a Bayesian method for the real-time characterization and forecasting of COVID-19 in California and regions of New Mexico; [8] analysed the dynamics of COVID-19 in Germany, by applying a novel combination of epidemiological modelling with specialized neural networks; [9] developed a Bayesian approach based on the probabilistic SIR model using data regarding daily confirmed cases of COVID-19 from six states in the United States; [10] studied the behaviour of COVID-19 in France, performing Bayesian estimation of the initially infected and the parameters of the compartmental SIRD model. A more sophisticated approach considers epidemiological models with time-dependent parameters in order to account for different control strategies, such as remote working from home, closure of schools, lockdowns, etc. For instance, [11] proposed an extended Markov SIR model and developed a R-routine with a calibration procedure that incorporates various types of time-varying quarantines for underreported infected cases in China; [12] proposed a calibration of the SEIR model to noisy data based on the negative binomial generalized additive model (GAM) for determining the time-varying effective contact rate and used this approach to fit the number of daily cases of COVID-19 in Ireland; [13] extended the parsimonious branching process model of the spread of disease and applied Bayesian inference to estimate the instantaneous reproduction number (*R*_*t*_) to explore the impact of heterogeneity on the distribution of secondary infections by COVID-19 in Ireland. On the other hand, the statistical models most applied for forecasting have been the ARIMA family, which predict future observations of a time series based on the linear dependence—deterministic or stochastic—of past [14–19]. In the search to improve both nowcasting and forecasting, different hybrid and ensemble approaches have been explored with accuracy results many times better than others; see for instance [20, 21]. However, although many models have been used in the analysis of Covid-19, to the best of our knowledge, there is no methodology that combines both the dynamics between populations and the dependence of past.

Motivated by the behaviour of COVID-19 in Colombia [22, 23], we propose a hybrid method to predict the daily values of infected and dead by COVID-19. Initially, the inverse problem of the SIRD model is solved by estimating the parameters via Bayesian inference through the Monte Carlo algorithm based on Markov Chains (MCMC), extending the work in [24]. In the estimation process we assign two prior distributions for each parameter and implement the selection criteria of Bayesian models: log of pseudo-marginal likelihood (LPML) [25] and observed information criterion (DIC3) [26], then we fit a seasonal ARIMA model to the residuals obtained from the discrepancy between the observed data and the best SIRD model selected.

The rest of the article is organized as follows. Section 2 presents the materials and methods, describes the data of the application, the hybrid and SIRD models, Bayesian estimation of its parameters and the comparison criteria of the Bayesian models. It also presents a simulation study that evaluates the statistical properties of the Bayesian estimators. Section 3 applies the method proposed to data from the cities of Calarcá and Pasto in Colombia and, finally, Section 4 presents the conclusions and suggestions for future research.

## 2 Materials and methods

### 2.1 The data

The data used in this work are the daily cases of people infected and dead by COVID-19 reported by Colombia’s National Health Institute (https://www.ins.gov.co/Paginas/Boletines-casos-COVID-19-Colombia.aspx) for the cities of Calarcá and Pasto. Specifically, information was used from 21 March (date when the first case was reported) to 28 July 2020 for Calarcá and from 27 March (date in which four cases had been reported) to 31 July 2020 for Pasto. Information on the total number of inhabitants (N) for both cities was obtained from the website of Colombia’s National Administrative Department of Statistics, (DANE), which for Calarcá is N = 74890 and for Pasto is N = 392589. In both cases, it was assumed that the number of recovered and of deaths at the start of the pandemic was zero.

### 2.2 The hybrid model

Let {*y*_*j*_, *j* = 1, …, *n*} be a realization of the stochastic process $\left\{Y_{t},t\in \tau \subset R\right\}$ and assume that the time series can be decomposed as

$$\begin{array}{c} y_{j}=f(X_{\theta }\left(t_{j}\right))+\epsilon_{j},\;j=1,2,\ldots ,n, \end{array}$$ (1)

where the term *f*(*X*_***θ***_(*t*_*j*_)) is called the signal and {*ϵ*_*j*_, *j* = 1, 2, …, *n*} are the errors. In many situations it is assumed that errors are independent and identically distributed $N(0,\sigma_{\epsilon }^{2})$. In this case, if *f*(*X*_***θ***_(*t*)) is a deterministic function then (1) is a regression model and observations are non-correlated [27]. The component *f*(*X*_***θ***_(*t*)) describes the trend of the observations, which can be linear, polynomial, exponential, logistic or harmonic polynomial, and so on. A special case occurs when *X*_***θ***_(*t*) is the solution of a mathematical model of the form

$$\begin{array}{c} \frac{dX_{\theta }\left(t\right)}{dt}=F\left(X_{\theta },t,\theta \right), \end{array}$$ (2)

and *f* is the identity, in this case the solution of (2) corresponds to the regressor of (1). Prior estimation of vector of parameters ***θ*** from model (2) is known as the inverse problem. On the other hand, as we said before, two common assumptions are the independence and normality of the random errors. However, autocorrelation, non-stationarity or stochastic seasonality can remain in the residuals. One way to correct the model is by fitting an appropriate model to the series {*ϵ*_*j*_, *j* = 1, 2, …, *n*}. In modelling the COVID-19 data, ARIMA and seasonal ARIMA (SARIMA) have been widely used due to their satisfactory forecasts. The *SARIMA*(*p*, *d*, *q*) × (*P*, *D*, *Q*)_*s*_ model for time series {*ϵ*_*j*_, *j* = 1, 2, …, *n*} is given by

$$\begin{array}{c} \phi_{p}\left(B\right)\Phi_{P}\left(B^{s}\right){\left(1-B\right)}^{d}{\left(1-B^{s}\right)}^{D}\epsilon_{j}=\delta_{q}\left(B\right)\Delta_{Q}\left(B^{s}\right)a_{j}, \end{array}$$ (3)

where *ϕ*_*p*_(*B*) and *δ*_*p*_(*B*) are called the regular autoregressive and moving average factors (polynomials), and Φ_*P*_(*B*^*s*^) and Δ_*Q*_(*B*^*s*^) are the seasonal autoregressive and moving average factors (polynomials), respectively. The roots of these polynomials lie outside of the unit circle, the series {*a*_*j*_} is a white noise $N(0,\sigma_{a}^{2})$ and *s* refers to the seasonal index; (1 − *B*)^*d*^ and (1 − *B*^*s*^)^*D*^ are two differencing factors of orders *d* and *D*. Generally, *d*, *D*, *p*, *P*, *q*, *Q* ∈ {0, 1}. If all orders are zero, then {*ϵ*_*j*_} = {*a*_*j*_}; otherwise, under model (3) errors *ϵ*_*j*_ can be generated by

$$\epsilon_{j}=\frac{\delta_{q}\left(B\right)\Delta_{Q}\left(B^{s}\right)}{\phi_{p}\left(B\right)\Phi_{P}\left(B^{s}\right){\left(1-B\right)}^{d}{\left(1-B^{s}\right)}^{D}}a_{j},$$

and the hybrid model is given by

$$\begin{array}{c} y_{j}=f(X_{\theta }\left(t_{j}\right))+\frac{\delta_{q}\left(B\right)\Delta_{Q}\left(B^{s}\right)}{\phi_{p}\left(B\right)\Phi_{P}\left(B^{s}\right){\left(1-B\right)}^{d}{\left(1-B^{s}\right)}^{D}}a_{j},\;j=1,2,\ldots ,n. \end{array}$$ (4)

### 2.3 The deterministic model

Accordingly to (2) and for data before vaccination, in this work we employ the classical SIRD model [2] where the population *N*(*t*) is further divided into: individuals who are susceptible *S*(*t*), infected *I*(*t*), recovered *R*(*t*) and dead *D*(*t*). Moreover, it is assumed that the population is homogeneous and remains constant over a given infectious period, i.e., *N* = *N*(*t*) = *S*(*t*) + *I*(*t*) + *R*(*t*) + *D*(*t*), ∀*t* ≥ *t*_0_, where *t*_0_ is the initial instant. The SIRD model, constraints to *S*(*t*_0_) = *N* − *I*_0_ − *D*_0_ − *R*_*e*_, is given by the following nonlinear system of differential equations:

$$\begin{array}{c} \begin{array}{cc} \frac{dS}{dt} & =-\left(\gamma +\phi \right)R_{0}\frac{I(t)S(t)}{N}\\ \frac{dI}{dt} & =\left(\gamma +\phi \right)R_{0}\frac{I(t)S(t)}{N}-(\gamma +\phi )I\left(t\right)\\ \frac{dR}{dt} & =\phi I(t)\\ \frac{dD}{dt} & =\gamma I(t), \end{array} \end{array}$$ (5)

where *I*(*t*_0_) = *I*_0_ > 0, *D*(*t*_0_) = *D*_0_ ≥ 0, and *R*(*t*_0_) = *R*_*e*_ ≥ 0 are the initial values of infected, dead and recovered individuals, respectively, *β* is the infection rate, *ϕ* is the recovery rate, *γ* is the mortality rate. $\tau =\frac{1}{\gamma +\phi }$ denotes the average time of the infectious period and *R*_0_ = *β*/(*ϕ* + *γ*) is the basic reproductive number. It is well known that if *R*_0_ > 1, the epidemic propagates and if *R*_0_ < 1, the epidemic subsides. It is important to note that the SIRD model is typically written in terms of the rates *β*, *γ* and *ϕ*. However we reparameterized it in terms of *R*_0_, *γ* and *ϕ* observing that *β* can be obtained through *ϕ*, *γ* and *R*_0_. This reparameterization was motivated by knowledge of the behavior of *R*_0_ for COVID-19.

### 2.4 Inverse problem solution

#### 2.4.1 Statistical model

Let **z** = [**y**; **d**]_*n*×2_ be a matrix whose columns **y** = (y_1_, …, y_*n*_)^⊤^ and **d** = (d_1_, …, d_*n*_)^⊤^ are two independent random vectors of n observations that follow the dynamics of the SIRD model with unknown parameters, where y_*j*_ and d_*j*_ represents, respectively, the number of infections and deaths reported between day *j* − 1 and *j*, with *j* = 1, …, *n*. This period of time is denoted by the interval (*t*_*j*−1_, *t*_*j*_]. Due to the nature of y_*j*_ and d_*j*_, their probabilistic behaviour is modelled through independent Poisson distributions, that is, ${\text{y}}_{j}∼\text{Poisson}\left({\text{y}}_{j}^{*}\left(\theta \right)\right)$ and ${\text{d}}_{j}∼\text{Poisson}\left({\text{d}}_{j}^{*}\left(\theta \right)\right)$, where

$$\begin{array}{c} \;{\text{y}}_{j}^{*}\left(\theta \right)=\int_{t_{j-1}}^{t_{j}}\frac{\left(\gamma +\phi \right)R_{0}S\left(t\right)I\left(t\right)}{N}dt\;\text{and}\;{\text{d}}_{j}^{*}\left(\theta \right)=\int_{t_{j-1}}^{t_{j}}\gamma I\left(t\right)dt, \end{array}$$ (6)

represent the mean number of new infections and deaths, respectively, caused by the disease during the time interval (*t*_*j*−1_, *t*_*j*_]. It is important to note from Eq (5) that the individuals are infected at a rate of $\frac{\left(\gamma +\phi \right)R_{0}S\left(t\right)I\left(t\right)}{N}$, which implies that the mean number of new cases in (*t*_*j*−1_, *t*_*j*_] can be obtained through $\int_{t_{j-1}}^{t_{j}}\frac{\left(\gamma +\phi \right)R_{0}S\left(t\right)I\left(t\right)}{N}dt$.

#### 2.4.2 Likelihood function

Let ***θ*** = (*R*_0_, *ϕ*, *γ*)^⊤^ be the vector of parameters in the SIRD model, the likelihood function of ***θ*** given the vector of observations **z**, is given by

$$\begin{array}{c} \begin{array}{cc} \text{L}(\theta |\text{z})=\text{L}(\theta |\text{y},\text{d}) & =\text{L}(\theta |\text{y})\text{L}(\theta |\text{d})\\ & =\prod_{j=1}^{n}\text{Poisson}\left({\text{y}}_{j}^{*}\left(\theta \right)\right)\times \text{Poisson}\left({\text{d}}_{j}^{*}\left(\theta \right)\right)\\ & =\prod_{j=1}^{n}\frac{({\text{y}}_{j}^{*}\left(\theta \right){)}^{{\text{y}}_{j}}e^{-{\text{y}}_{j}^{*}(\theta )}({\text{d}}_{j}^{*}\left(\theta \right){)}^{{\text{d}}_{j}}e^{-{\text{d}}_{j}^{*}(\theta )}}{{\text{y}}_{j}!{\text{d}}_{j}!} \end{array} \end{array}$$ (7)

Our solution of the inverse problem seeks to first estimate the vector of parameters ***θ*** of the model (5) from the number of infections and deaths reported in a population during a period of time and then solve the SIRD model using those values. Specifically, we propose to perform the estimation process of ***θ*** from a Bayesian approach through MCMC algorithms.

#### 2.4.3 Bayesian inference

*2.4.3.1 Prior and posterior distributions*. We define non-informative priors for *ϕ* and *γ* and, for *R*_0_ we use two proposals: i) a prior distribution that takes into account the information available about this parameter from the Colombian National Health Institute and ii) an exponential distribution. Specifically, we allocate *ϕ* ∼ U(0, 1), *γ*|*ϕ* ∼ *U*(0, 1 − *ϕ*) because the average duration of the infectious period (*τ*) must be greater than one day, which implies the relation *ϕ* + *γ* < 1; under i) *R*_0_ ∼ U(*a*_1_, *b*_1_) and under ii) *R*_0_ ∼ exp(*a*).

Therefore, the joint prior distribution of vector ***θ*** can be obtained through *π*(***θ***) = *π*(*R*_0_) × *π*(*ϕ*) × *π*(*γ*|*ϕ*), which can be rewritten under i) as *π*_1_(***θ***) = U(*a*_1_, *b*_1_) × U(0, 1) × U(0, 1 − *ϕ*) and under ii) as *π*_2_(***θ***) = exp(*a*) × U(0, 1) × U(0, 1 − *ϕ*).

The estimation procedure consists of simulating values of the posterior distribution of ***θ***, which is obtained by combining the prior distribution *π*_*i*_(***θ***), *i* = 1, 2, with the likelihood function (7); that is

$$\begin{array}{c} \pi (\theta |\text{z})\propto \pi (\theta )\times \text{L}(\theta |\text{z}). \end{array}$$ (8)

However, due to the complexity of this process, caused by the dependence of this distribution on the solution of a system of ODE, we implement the t-walk algorithm [28] through the R package Rtwalk, since it is particularly well suited for generating samples from a posteriori distributions using nonstandard models.

This algorithm generates samples from continuous distributions starting at two independent random points in the sample space from there each move is generated from one of four proposed distributions (see the options in [28]) and accepted with a probability as in Metropolis-Hastings on the product space, which implies that two chains are not generated independently of each other. The algorithm can be summarized as follows: i) two points are randomly and independently selected in the sample space; ii) then with probability of 0.5 the value that will be updated is selected; iii)subsequently, the proposed distribution is randomly selected (among four possible ones) and a new value is generated while the other remains unchanged and iv) this value will be accepted through calculation of the acceptance probability of the Metropolis-Hastings algorithm.

Finally, the implemented algorithm can be summarized as follows:

1) Assign initial values to the number of susceptible, infected, recovered and dead individuals, i.e., *S*(0) = *s*_0_, *I*(0) = *i*_0_, *R*(0) = *r*_0_ and *D*(0) = *d*_0_.
2) Randomly and independently generate two initial values for each of the parameters of the SIRD model: *R*_0_, *ϕ* and *γ*.
3) Numerically solve the ODE system in (5) and subsequently the integrals in (6).
4) Once the integrals have been determinated numerically in step 3), values of the a posteriori distribution *π*(*θ*|**z**) are generated.
5) Return to step 2) and repeat the process until convergence is obtained.

Upon obtaining the results from two chains, 250000 samples, called burn-in, are discarded, and 50000 more samples (with spacing of 300) are used to calculate posterior summaries.

Convergence was monitored through the statistical diagnostic *potential scale reduction factor* (psrf) proposed by Gelman and Rubin [29]. The psrf denoted by $\hat{R}$ allows monitoring the convergence to the target distribution (posterior distribution in this case) of two randomly initialized chains calculating the ratio between the average of the variances obtained in each chain and the variance of the grouped chains; if the chains have reached the target distribution, this ratio will be close to 1 (in this work $\hat{R}<1.2$ was used), otherwise $\hat{R}$ will be far from 1.

#### 2.4.4 Bayesian model selection

To select the model that best fits the COVID-19 data in each city analysed, the Bayesian criteria for model selection called log pseudo-marginal likelihood (LPML) [30] and a modified version of the DIC, named DIC_3_ [26], were implemented.

The LPML summarizes the conditional predictive ordinate (CPO), obtained from the posterior predictive distribution. For the *j*-th observation the CPO, denoted by CPO_*j*_, is expressed as ${\text{CPO}}_{j}=\int_{\Theta }f\left({\text{z}}_{j}|\theta \right)\pi \left(\theta |D^{\left(-j\right)}\right)d\theta$ where *f*(**z**_*j*_|***θ***) is the probability mass function of **z**_*j*_ obtained as the product between $\text{Poisson}({\text{y}}_{j}^{*}\left(\theta \right))$ and $\text{Poisson}({\text{d}}_{j}^{*}\left(\theta \right))$, D denotes the full dataset, $D^{\left(-j\right)}$ the data without the *j*−th observation and *π*(***θ***|⋅) is the posterior distribution from ***θ***. Because of the lack of a closed form for this integral, the CPO_*j*_ is estimated through a harmonic-mean approximation as proposed by [31] through ${\hat{CPO}}_{j}=\{\frac{1}{Q}\sum_{q=1}^{Q}\frac{1}{f({\text{z}}_{j}|\theta_{q})}{\}}^{-1}$, where ***θ***_1_, …, ***θ***_*Q*_ is a post burn-in sample of size *Q* from π(θ|D). Finally the LPML is calculated as $\text{LPML}=\sum_{j=1}^{n}\text{log}\left({\hat{CPO}}_{i}\right)$. The best fit is given by the largest LPML.

Although other criteria can be used, in this work we implemented an alternative to DIC [32], named DIC_3_ [26], due to the complexity of our model since the parameters of the Poisson distributions are obtained from the solution of a system of ODE. This criterion is defined as ${\text{DIC}}_{3}=\bar{D(\theta )}+\tau_{D}$, $\bar{D(\theta )}=-2\text{E}\left\{\text{log}\left[f\left(\text{z}|\theta \right)\right]|\text{z}\right\}$, where *f*(**z**|***θ***) is the likelihood function presented in (7), E{log[*f*(**z**|***θ***)]|**z**} is the posterior expectation of log[*f*(**z**|***θ***)] and *τ*_*D*_ is a measure of the effective number of parameters in the model, calculated as $\tau_{D}=\bar{D(\theta )}+2\;\text{log}\left(\text{E}\left[f\left(\text{z}|\theta \right)|\text{z}\right]\right)$. Thus, DIC_3_ = −4E{log[*f*(**z**|***θ***)]|**z**} + 2 log(E[*f*(**z**|***θ***)|**z**]). Following [26] the first term in the DIC_3_ definition can be approximated by $-4\bar{D}=-4\frac{1}{Q}\sum_{q=1}^{Q}\sum_{i=1}^{n}\text{log}[f({\text{z}}_{i}|\theta^{\left(q\right)})]$ and the second term by $\sum_{i=1}^{n}2\;\text{log}\;\hat{f}\left({\text{z}}_{i}|\theta \right)$ with $\hat{f}\left({\text{z}}_{i}|\theta \right)=\frac{1}{Q}\sum_{q=1}^{Q}f\left({\text{z}}_{i}|\theta^{\left(q\right)}\right)$. The best fit is given by the lowest DIC_3_.

### 2.5 Simulation study

In this section, a simulation study is conducted in order to assess the statistical properties of the Bayesian estimators of the SIRD model parameters. Initially, we set *N* = 10000 and *S*(0) = 9999, *I*(0) = 1, *R*(0) = 0, *D*(0) = 0 as initial conditions; then, we consider the three hypothetical parameter vectors ***θ***_1_ = (1.1, 0.7, 0.2)^⊤^, ***θ***_2_ = (1.5, 0.3, 0.1)^⊤^ and ***θ***_3_ = (3, 0.08, 0.02)^⊤^, for two sample sizes, *n* = 30 and *n* = 60. Under these initial conditions, the population *N* and each combination of ***θ*** and *n*, we generate 100 samples of **z** denoted by **z**_*l*_, *l*: 1, …, 100. Each sample **z**_*l*_ is generated from ${\text{y}}_{lj}∼\text{Poisson}({\text{y}}_{j}^{*}\left(\theta \right))$ and ${\text{d}}_{lj}∼\text{Poisson}({\text{d}}_{j}^{*}\left(\theta \right))$, *j* = 1, …, *n*, where ${\text{y}}_{j}^{*}\left(\theta \right)$ and ${\text{d}}_{j}^{*}\left(\theta \right)$ are obtained from (6).

From each **z**_*l*_, the parameters are estimated from the posterior distribution associated with ***θ***, *π*(***θ*** ∣ **z**_*l*_), using in (8) the joint prior distribution *π*(***θ***) = U(1, 5) × U(0, 1) × U(0, 1).

For each parameter in ***θ***, two Markov chains of 30000 size are generated and their convergence is evaluated through the psrf ($\hat{R}<1.2$), as mentioned previously. After discarding a burn-in of 15000 values and applying a lag of 20, the posterior mean is estimated. We also estimate the relative bias (RB) of ${\hat{\theta }}_{r}$, *r* = 1, 2, 3 given by $\text{RB}=\frac{1}{100}\sum_{l=1}^{100}({\hat{\theta }}_{r_{l}}/\theta_{r}-1)$ and the mean square error, $\text{MSE}=\frac{1}{100}\sum_{l=1}^{100}({\hat{\theta }}_{r_{l}}-\theta_{r}{)}^{2}$, where ${\hat{\theta }}_{r_{l}}$ is the estimator of *θ*_*r*_ obtained with the *l-th* simulated sample, **z**_*l*_.

Tables 1 and 2 summarize the results for *n* = 30 and *n* = 60, respectively. Notice that (i) the MSE decreases with the sample size increases; (ii) both RB and MSE are reasonably small, suggesting a good precision and (iii) the estimator of *R*_0_ has the best accuracy and precision. So, based on these simulation results, it can be concluded that Bayesian estimators of the SIRD model parameters have satisfactory statistical properties, which improve as sample size increases.

**Table 1:** True values of parameters, and posterior mean, RB, MSE and 95% credible intervals of parameter estimates of the SIRD model for *n* = 30.

*n* = 30							
	True		Post. mean	RB	MSE	Quantile	
						2.5%	97.5%
***θ***_1_	*R*_0_	1.1	1.1096	0.0088	0.0021	1.0581	1.2337
	*ϕ*	0.7	0.7728	0.1040	0.0320	0.3934	0.9589
	*γ*	0.2	0.1410	-0.2947	0.0161	0.0295	0.4457
***θ***_2_	*R*_0_	1.5	1.5138	0.0092	0.0101	1.3831	1.7330
	*ϕ*	0.3	0.3258	0.0862	0.0065	0.1738	0.4468
	*γ*	0.1	0.0767	-0.2324	0.0016	0.0331	0.1378
***θ***_3_	*R*_0_	3	2.9572	-0.0142	0.9828	1.4665	4.7788
	*ϕ*	0.08	0.0948	0.1852	0.0037	0.0063	0.2308
	*γ*	0.02	0.0213	0.0663	0.0005	0.0037	0.0861

**Table 2:** True values of parameters, and posterior mean, RB, MSE and 95% credible intervals of parameter estimates of the SIRD model for *n* = 60.

*n* = 60							
	True		Post. mean	RB	MSE	Quantile	
						2.5%	97.5%
***θ***_1_	*R*_0_	1.1	1.1005	0.0004	2.9e-05	1.0893	1.1121
	*ϕ*	0.7	0.7538	0.0769	0.0062	0.6391	0.8762
	*γ*	0.2	0.1445	-0.2770	0.0046	0.0747	0.2413
***θ***_2_	*R*_0_	1.5	1.4988	-0.0007	0.0002	1.4756	1.5220
	*ϕ*	0.3	0.3124	0.0413	0.0010	0.2245	0.3651
	*γ*	0.1	0.0888	-0.1112	0.0010	0.0463	0.1805
***θ***_3_	*R*_0_	3	3.2138	0.0712	0.6397	1.8240	4.8917
	*ϕ*	0.08	0.0563	-0.2959	0.0014	0.0027	0.1099
	*γ*	0.02	0.0177	-0.1144	0.0005	0.0007	0.0914

## 3 Results and discussion

This section reports the application of the hybrid method proposed to predict the daily notifications of infected and dead due to COVID-19 in two Colombian cities: Calarcá and Pasto. Initially, the SIRD model is estimated and fitted to the data, then a *SARIMA*(*p*, *d*, *q*) × (*P*, *D*, *Q*)_*s*_ is fitted to the residuals $\left\{\hat{\epsilon_{j}}\right\}$ to calculate the predictions of the hybrid model (4).

### 3.1 Covid-19 predictions for Calarcá—Colombia

For Calarcá we considered the following initial conditions: population size *N* = 74890, since the first infection was notified on 21 March 2020, so *I*(0) = 1, *S*(0) = 74889, *R*(0) = 0 and *D*(0) = 0. The estimation of the SIRD model parameters was based on two joint prior distributions: *π*_1_(***θ***) = U(1, 3) × U(0, 1) × U(0, 1) and *π*_2_(***θ***) = exp(1) × U(0, 1) × U(0, 1). In joint distribution *π*_1_(***θ***), the uniform prior distribution *U*(1, 3) for *R*_0_ was chosen based on the fact that the Colombia’s National Health Institute established that at the beginning of the pandemic 1 < *R*_0_ < 3 [33]. In the joint distribution *π*_2_(***θ***), an exponential distribution with parameter 1 was chosen to make sure the parameter was positive. For each parameter vector we generated two Markov chains of size 100000 from the posterior distribution *π*_*i*_(***θ*** ∣ **z**), *i* = 1, 2, and convergence of chains was monitored using the psrf ($\hat{R}<1.2$), as mentioned previously. Then, a burn-in of 50000 and a lag of 20 were applied on the chains to calculate the posterior mean. In order to select the best posterior mean we calculated the LPML and DIC3 selection criteria for posterior distributions obtained with *π*_1_(***θ***) and *π*_2_(***θ***) priors, and values are given in Table 3. Notice that there is no significant difference between the two models, so we can use either of them. In this case we prefer *π*_1_(***θ*** ∣ **z**). Table 4 shows the posterior means, standard deviations, and 95% CI based on quantiles 0.025 and 0.975 of SIRD model parameters for Calarcá data. We can conclude that at the beginning of pandemic i) on average, one infected person could infect approximately 1.1 susceptible people during the infectious period; ii) for every 1000 individuals infected, 126 recovered per day, 2.8 died due to the disease and the others continued infected; iii) the average time of duration of the infectious period, *τ*, is approximately eight days; iv) with 0.95 of probability: a) the number of individuals infected by an infected person varied between 1.096 and 1.122; b) for each 1000 infected individuals, between 113 and 135 people recovered daily and between 0.08 and 9.2 individuals died daily due to COVID-19.

**Table 3:** Selection criteria of Bayesian models for Calarcá.

Model	LPML	DIC3
*π*_1_(***θ*** ∣ **z**)	-119.490	236.114
*π*_2_(***θ*** ∣ **z**)	-120.134	235.822

**Table 4:** Bayesian estimations of the parameters of the SIRD model for Calarcá.

Parameter	Post. mean	$\hat{\sigma }$	Quantile	
			2.5%	97.5%
*R*_0_	1.107	0.006	1.096	1.122
*ϕ*	0.126	0.005	0.113	0.135
*γ*	0.0028	0.002	0.00008	0.0092

**Fig 1:**
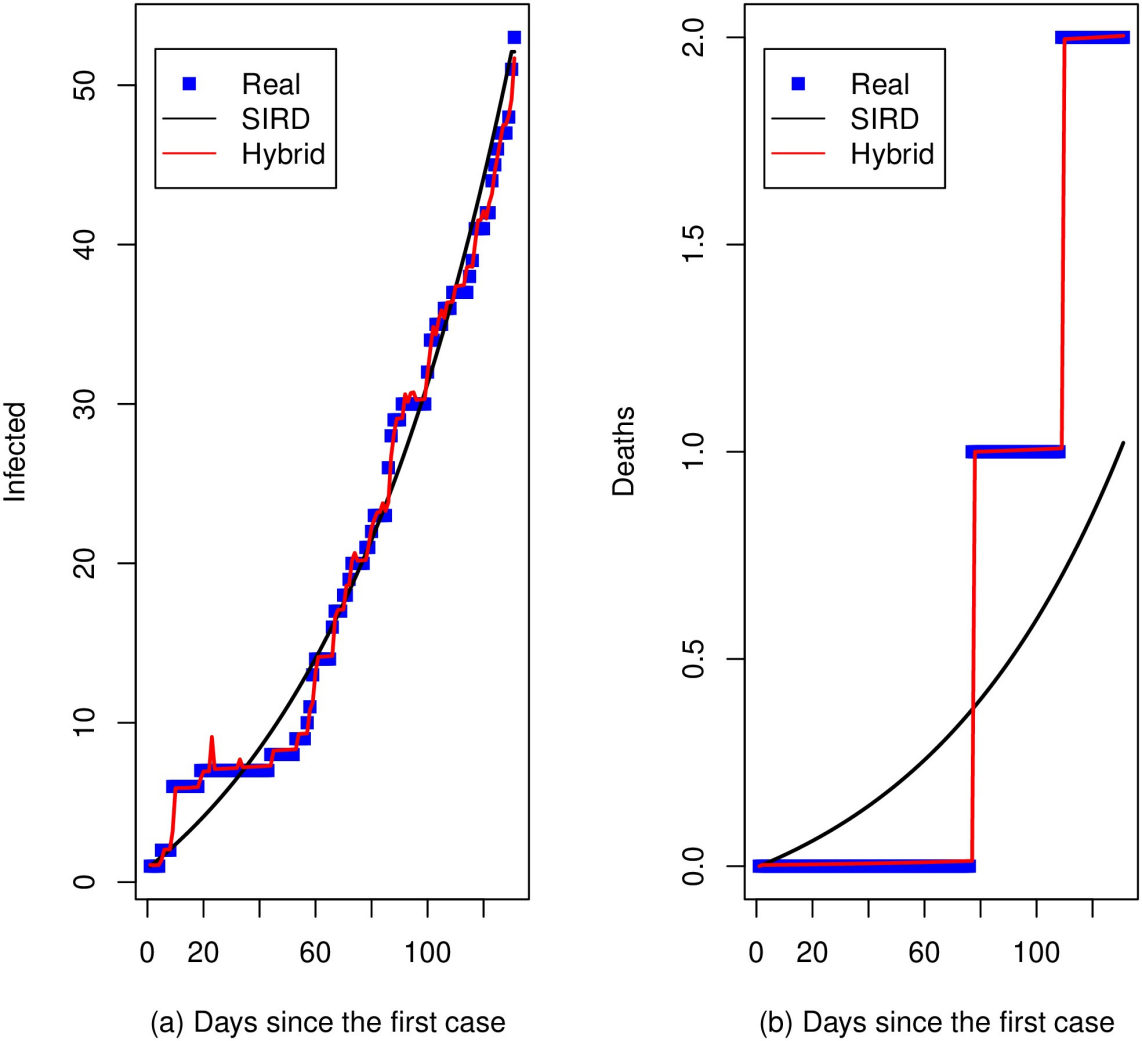
Real data (blue squares), predictions with the SIRD model (black curves) and SIRD + SARIMA hybrid (red lines), Calarcá—Colombia. (a) Days since the first case. (b) Days since the first case.

Fig 1(a) and 1(b) show respectively, the cumulative number of infections and deaths (blue squares) that were notified by the [34] between 20 March and 28 July 2020 (training set) with the predictions (black curves) from the respective estimated SIRD model. Red curves represent the predictions from the hybrid model with a *SARIMA*(1, 0, 0) × (1, 0, 0)_14_ for residuals of infected data and an *AR*(1) for residuals of death data. For time series of infected, the RMSE(*Hybrid*) = 0.63 and RMSE(*SIRD*) = 1.99 for the training set. For the prediction two-weeks ahead (test set) the RMSE(*Hybrid*) = 21.96 and RMSE(*SIRD*) = 21.35. The white-noise estimated variance is ${\hat{\sigma }}_{a}^{2}=0.425$. For the series of deaths, the RMSE(*Hybrid*) = 0.12 and RMSE(*SIRD*) = 0.56 for the training set. For the prediction two-weeks ahead (test set), the RMSE(*Hybrid*) = 1.02 and RMSE(*SIRD*) = 1.64. The white-noise estimated variance is ${\hat{\sigma }}_{a}^{2}=0.015$. Notice that in general the RMSE value is lowest in the hybrid model.

### 3.2 Covid-19 predictions for Pasto—Colombia

In this case the population size is *N* = 392589 and since there were four initially notified cases on 27 March 2020, the initial conditions are *I*_0_ = 4, *S*(0) = 392585, *I*(0) = 4, *R*(0) = 0 and *D*(0) = 0. We used the same priors as above and calculated the LPML and DIC3 criteria to select the best posterior distribution. According to the results in Table 5 the posterior *π*_1_ (***θ*** ∣ **z**) is selected as the best model because it has the highest LPML and lowest DIC3.

**Table 5:** Selection criteria of Bayesian models for Pasto.

Model	LPML	DIC3
*π*_1_(***θ*** ∣ **z**)	-979.54	1957.62
*π*_2_(***θ*** ∣ **z**)	-1187.62	2140.82

Table 6 shows the Bayesian estimations, accordingly to which we can conclude that at the beginning of the pandemic i) on average, for each 1000 infected individuals daily 71 recovered, three died, and the others continued infected; ii) the average duration of the infectious period, *τ*, is 13 days; iii) on average, an infected person can infect 1.6 susceptible people during the infectious period; iv) with a probability of 0.95, a) the number of susceptible individuals that can be infected by an infected person varies between 1.5 and 1.9; b) for each 1000 individuals infected, between 53 and 92 recovered daily, and the others died due to the infection or remained infected; c) for every 1000 infected individuals with COVID-19, between 2.6 and 3.8 died, the other individuals recovered or remained infected.

**Table 6:** Bayesian estimations of the parameters of the SIRD model for Pasto.

Parameter	Post. mean	$\hat{\sigma }$	Quantile	
			2.5%	97.5%
*R*_0_	1.659	0.0918	1.494	1.879
*ϕ*	0.071	0.0090	0.053	0.092
*γ*	0.0030	0.0003	0.0026	0.0038

**Fig 2:**
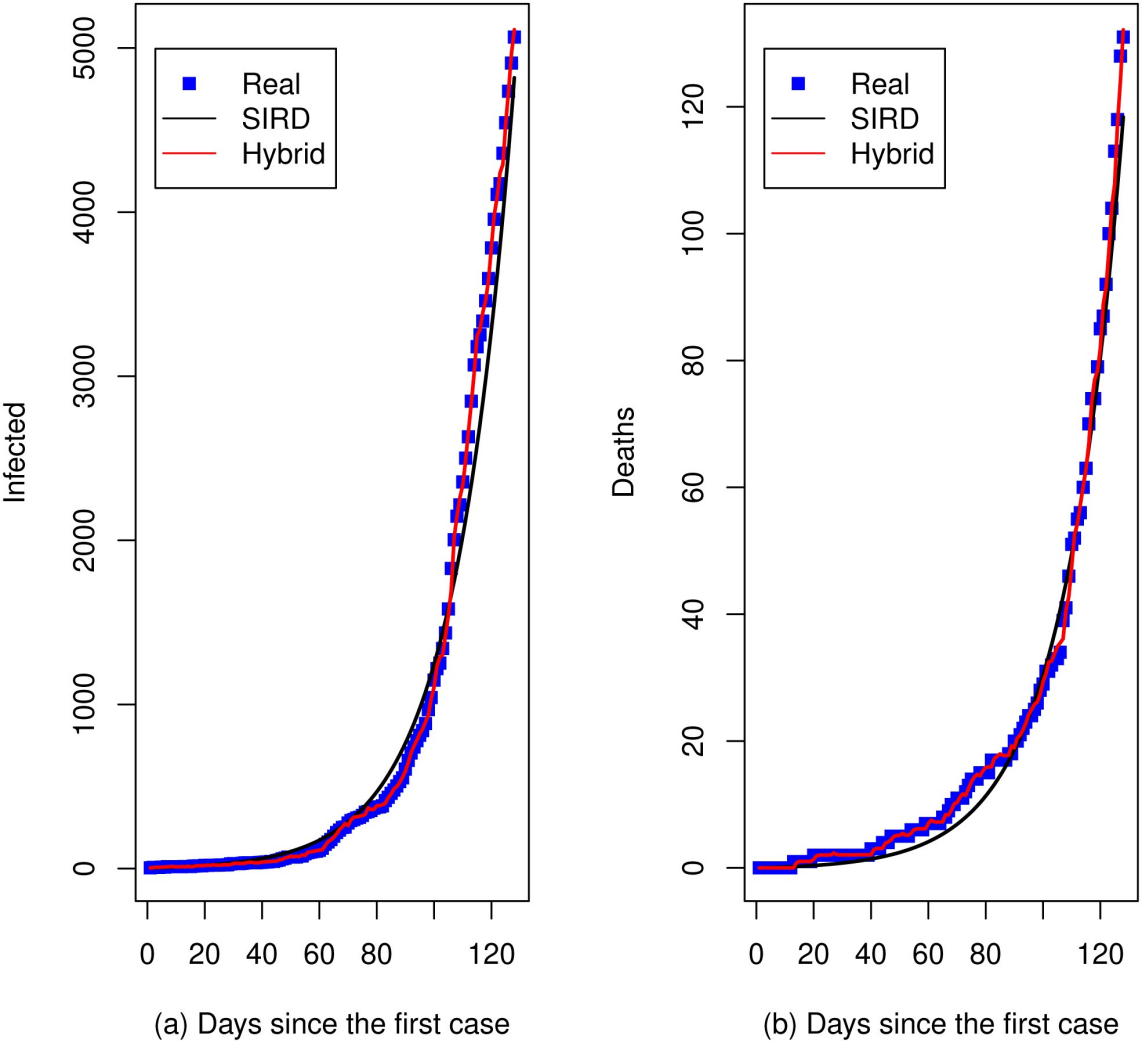
Real data (blue squares) and predictions with the SIRD model (black curves) and the *SIRD* + *SARIMA* hybrid (red curves), Pasto-Colombia. (a) Days since the first case. (b) Days since the first case.

Fig 2(a) and 2(b) show respectively, the cumulative number of infections and deaths (blue squares) that were notified by the [34] for the first 128 days of the pandemic between 28 March and 31 July 2020. Black curves represent the predictions from the respective estimated SIRD model and red curves represent the predictions from the respective hybrid model where a *SARIMA*(1, 1, 1) × (1, 0, 0)_7_ was fitted to the residuals of the mathematical model for infected series and a *SARIMA*(1, 1, 0) × (1, 0, 0)_7_ for the residuals of the mathematical model for death series. For time series of infected, the RMSE(*Hybrid*) = 21.4 and RMSE(*SIRD*) = 191.4 for the training set. For the prediction two-weeks ahead (test set), the RMSE(*Hybrid*) = 133.2 and RMSE(*SIRD*) = 1195.1. The white-noise estimated variance is ${\hat{\sigma }}_{a}^{2}=472.5$. For the series of deaths, the RMSE(*Hybrid*) = 1.06 and RMSE(*SIRD*) = 3.34 for the training set. For the prediction two-weeks ahead (test set), the RMSE(*Hybrid*) = 6.57 and RMSE(*SIRD*) = 10.63. The white-noise estimated variance is ${\hat{\sigma }}_{a}^{2}=1.15$. In all cases, the RMSE is much lower in the hybrid approach.

It is important to highlight that the average times of transmission from an infected person, estimated as a function of $\hat{\phi }$ and $\hat{\gamma }$ in the SIRD model in the cities of Calarcá and Pasto, are close to those reported in prior studies. For instance, [35] found that for 30 provinces in China and 15 cities in the province of Hubei, this time varied from 7 to 14 days, and according to the Center for Coordination of Sanitary Alerts and Emergencies [36], it was determined that the COVID-19 infectious period was between 9 and 17 days. These results agree with our estimations of *τ* for Pasto and Calarcá of 8 and 13 days, respectively. Moreover, our estimations of *R*_0_ for both cities are also close to those reported by [37], that in some European countries 1.4 < *R*_0_ < 6.49 with a mean value of 3.28, while in Colombia, the National Health Institute reported an *R*_0_ < 3 indicating that our estimations of 1.107 and 1.659 for Calarcá and Pasto, respectively, are reasonable from the epidemiological point of view.

## 4 Conclusions

The great challenge that the COVID-19 pandemic has caused in the search for models that provide good predictions of the number of infected, dead and recovered, in the short- and medium-term is well known. These studies have enabled government entities to develop actions that minimize the viral contagion speed and to reduce the economic and social impacts on the population.

Motivated by this challenge, we propose a hybrid method based on the combination of the SIRD compartmental model and a SARIMA model. The SIRD model captures the series trend and takes into account the interactions among the distinct states of the population, while the SARIMA model incorporates stochastic components such as non-stationarity, autocorrelation and seasonality that are remaining in the residuals. We use our proposal to predict the behaviour of the series of infections and deaths due to COVID-19 in two Colombian cities, obtaining better performance, in the sense that it significantly diminished the values of RMSE, compared with the fit of only the SIRD model. In spite of being used with these data, the hybrid method described in this work can be applied to predict other infectious diseases.

Future studies that can be derived from this work include: (i) modelling both the number of infections and number of deaths through the negative binomial distribution and comparing the results obtained with the Poisson distribution used in this work; (ii) evaluating the effect of climatic, social and economic variables, for instance, on the parameters of the SIRD model; and (iii) extending the method proposed to other compartmental models that permit incorporating information about control measures, like those taken by different governments to reduce virus propagation.

## Supporting information

S1 File (ZIP)
