# Supplementary figures and images for: Hybrid prediction of infections and deaths due to COVID-19 in two Colombian data series
Source: PLoS One. 2023 Jun 8;18(6):e0286643. doi: 10.1371/journal.pone.0286643 (PMC10249875; doi:10.1371/journal.pone.0286643)

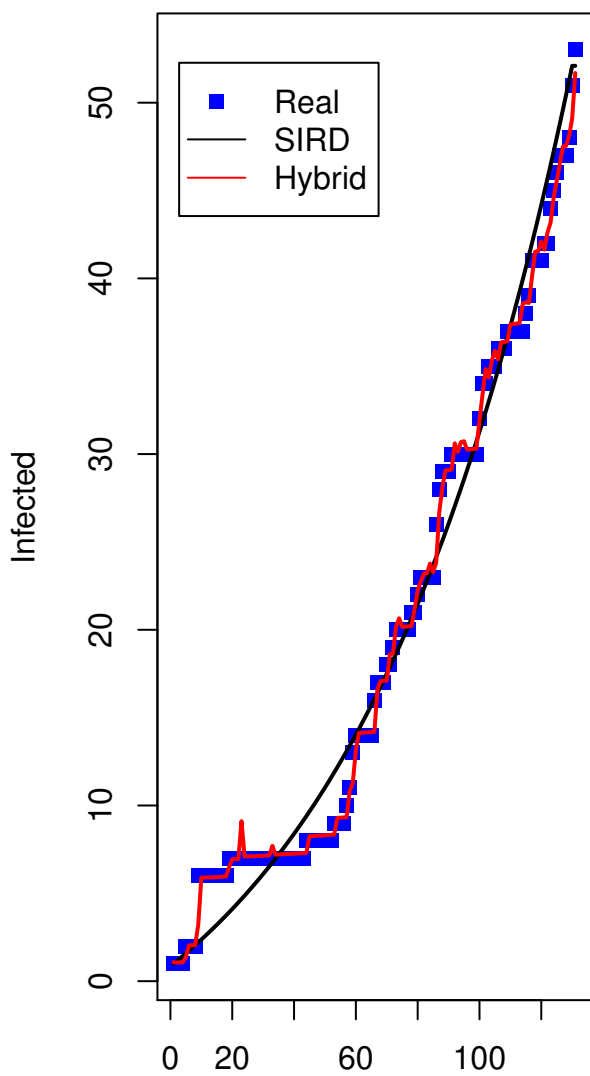

(a) Days since the first case

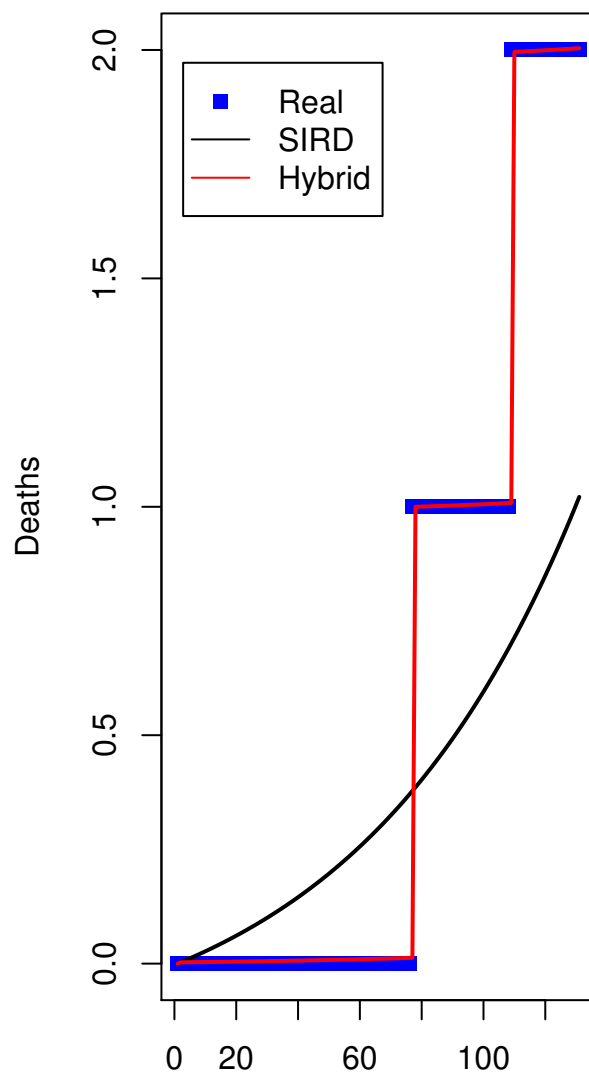

(b) Days since the first case

Supplement: S1 File — (ZIP) [file pone.0286643.s001.zip › Fig 1.pdf]

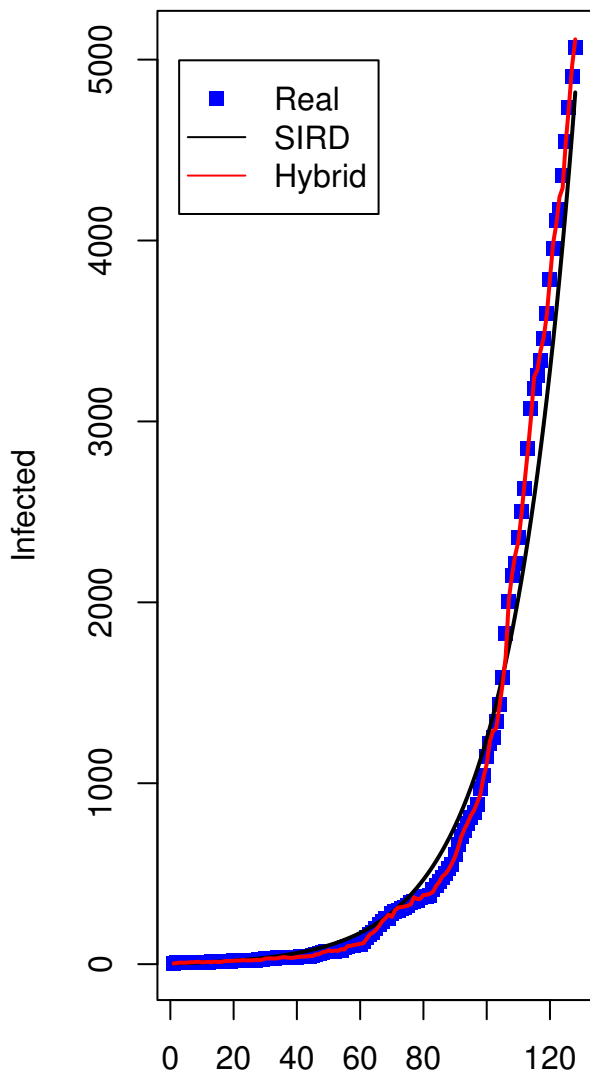

(a) Days since the first case

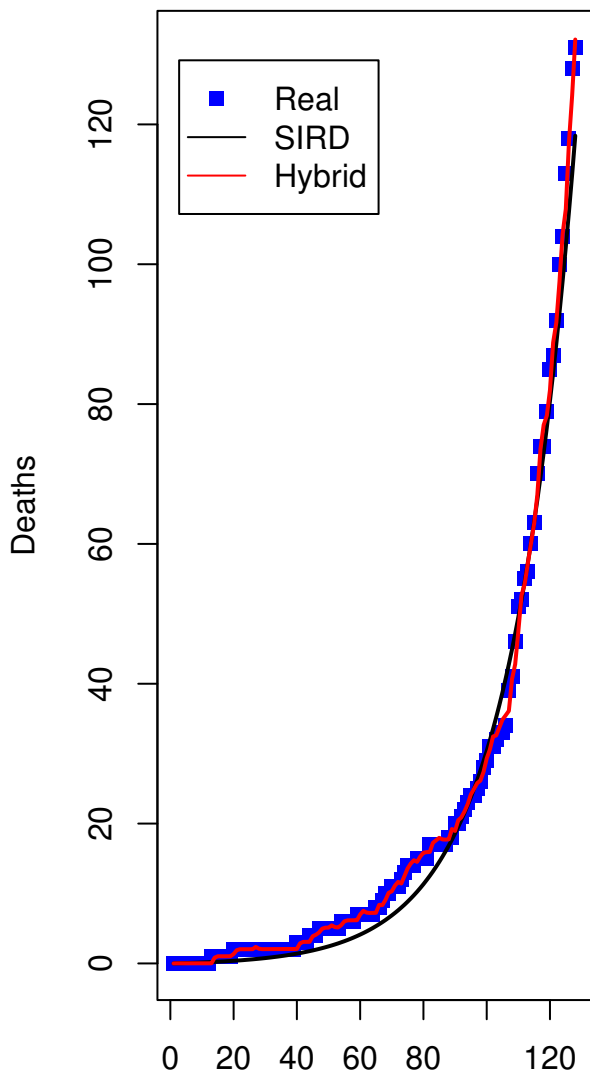

(b) Days since the first case

Supplement: S1 File — (ZIP) [file pone.0286643.s001.zip › Fig2.pdf]
